# Initial Results From an Everyday Memory and Metacognitive Intervention for Older Adults

**DOI:** 10.1093/geroni/igaa057.1617

**Published:** 2020-12-16

**Authors:** Ann Pearman, Christopher Hertzog, Emily Lustig, MacKenzie Hughes

**Affiliations:** Georgia Institute of Technology, Atlanta, Georgia, United States

## Abstract

We report results from a new intervention study implementing an Everyday Memory and Metacognitive Intervention (EMMI). This intervention trains older adults on self-regulatory procedures for achieving everyday life goals by implementing a metacognitive perspective where participants learn mindful control over life tasks that place demands on planning and memory (e.g., learning new names and managing prospective memory demands). Fifty-three participants, age range 65 to 83, were assigned to either the EMMI treatment group (n = 32, mean age = 70.13, SD = 3.2) or a waitlist control group (n = 21, mean age = 71.76, SD = 4.7). Individuals with probable memory impairments, as indexed by low MOCA scores, were excluded from the study. Outcomes included daily diary reports of everyday memory errors and a prospective memory telephone task. EMMI participants had fewer reported memory errors per day (M = 0.42) than controls (M = 0.64), one-tailed p = .03. EMMI participants also performed better than controls on the telephone task outcome variables: total number of phone calls completed and mean absolute deviation of call times from scheduled times for successfully completed calls (ps<.001). Subjective outcomes, including personal memory beliefs, life satisfaction, and perceived stress, showed greater pretest-posttest improvement in the EMMI group compared to the control group. This study is a successful initial demonstration of the efficacy of our intervention for improving everyday cognition in older adults and highlights the possibility of improving success in memory-demanding everyday life contexts, thereby contributing to resilient aging in an older population.

